# Application of engineered extracellular vesicles to overcome drug resistance in cancer

**DOI:** 10.3389/fonc.2022.1070479

**Published:** 2022-12-15

**Authors:** Taichiro Nonaka

**Affiliations:** ^1^ Department of Cellular Biology and Anatomy, Louisiana State University Health Sciences Center, Shreveport, LA, United States; ^2^ Feist-Weiller Cancer Center, Louisiana State University Health Shreveport, Shreveport, LA, United States

**Keywords:** extracellular vesicle, exosome, engineered EV, drug delivery, cancer

## Abstract

Targeted therapies have significantly improved survival rates and quality of life for many cancer patients. However, on- and off-target side toxicities in normal tissues, and precocious activation of the immune response remain significant issues that limit the efficacy of molecular targeted agents. Extracellular vesicles (EVs) hold great promise as the mediators of next-generation therapeutic payloads. Derived from cellular membranes, EVs can be engineered to carry specific therapeutic agents in a targeted manner to tumor cells. This review highlights the progress in our understanding of basic EV biology, and discusses how EVs are being chemically and genetically modified for use in clinical and preclinical studies.

## Introduction

Budding from the membranes of prokaryotic and eukaryotic cells, extracellular vesicles (EVs) act as intercellular messengers (1). They carry information in the form of proteins, lipids, RNA, and DNA to cells in the local environment and at distant sites, and retain many of the features of their parental cell of origin. Such information imprinting means that EVs can regulate processes during normal homeostasis; conversely, deregulation of EV function can translate to pathology in EV-targeted cells. This is exemplified by EV-dependent perturbations of the immune response, organ development, reproduction, and vasculogenesis (2, 3). EVs can also modulate the tumor microenvironment, leading to either enhanced or reduced tumorigenesis (4, 5). By understanding the fundamental biological processes that govern EV behavior, we will be able to exploit EVs to use them as conduits of anticancer therapeutics. By definition EVs are biocompatible, and researchers in the field are now fine-tuning EVs *via* chemical and genetic strategies to transform them into drug delivery systems (6). The pros and cons of EVs in the context of cancer therapy is the main focus of this review. We draw upon examples from basic and translational research to highlight the advantages and limitations of EV use in the preclinical and clinical settings. Our aim is to provide a conceptual framework to spur on novel research approaches in the EV field, with the ultimate aim of improving survival rates in cancer patients.

## Biochemical properties of EVs

EV secretion has been observed in virtually all kingdoms of life. EV biogenesis and cargo loading are tightly linked processes, and these steps are strictly regulated to ensure that the subsequent interaction with target cells produces the desired biological effects (7). Cargos as diverse as proteins, lipids, nucleic acids, cytokines and even organelles provide an information-rich payload that can be exploited to achieve downstream cellular effects (3). Indeed, the ExoCarta database contains 9,690 proteins, 3,300 mRNAs, and 1,010 different types of lipids that have been identified in exosomes, which underscores the complexity and heterogeneity of these nanostructures (8). Specific examples of select cargos are discussed below.

Small non-coding RNAs have been found within EVs, and may control molecular events in recipient cells by regulating the activity of target mRNAs, for example (9). These RNA cargos could be exploited as potential non-invasive biomarkers for multiple disorders such as those that affect the immune system (10). EV has been isolated from most cell types and body fluids, including saliva. Saliva diagnostics is a rapidly expanding field and the non-invasive saliva testing could greatly facilitate the early diagnosis of many diseases, including cancers (11, 12).

The lipid composition of EVs is very similar to that of their parental cells; this provides an ideal physical barrier to protect internal cargo from premature degradation. In addition to providing EV integrity, the large amounts of sphingomyelin and cholesterol prevent EV cargos from degradation by both nucleases and proteases (13). Such lipids also act as physiological buffers that ensure the pH and osmotic balance within EVs is maintained (14, 15). These properties could be exploited to effectively increase the amount of drug that is delivered to a particular tumor.

A suite of fatty acids in EVs provide the building blocks for the generation of signaling intermediates that regulate multiple physiological processes (16). Consistent with the coordinated regulation of EV biogenesis and function, the EVs also contain the enzymes required for conversion of fatty acids into bioactive products such as esterified fatty acids and eicosanoids (17). These features underlie the ability of EVs to regulate inflammatory processes involved in chronic tissue remodeling, cancer, asthma, rheumatoid arthritis and autoimmune disorders (18).

## Strategies to functionalize EVs

**Figure 1:**
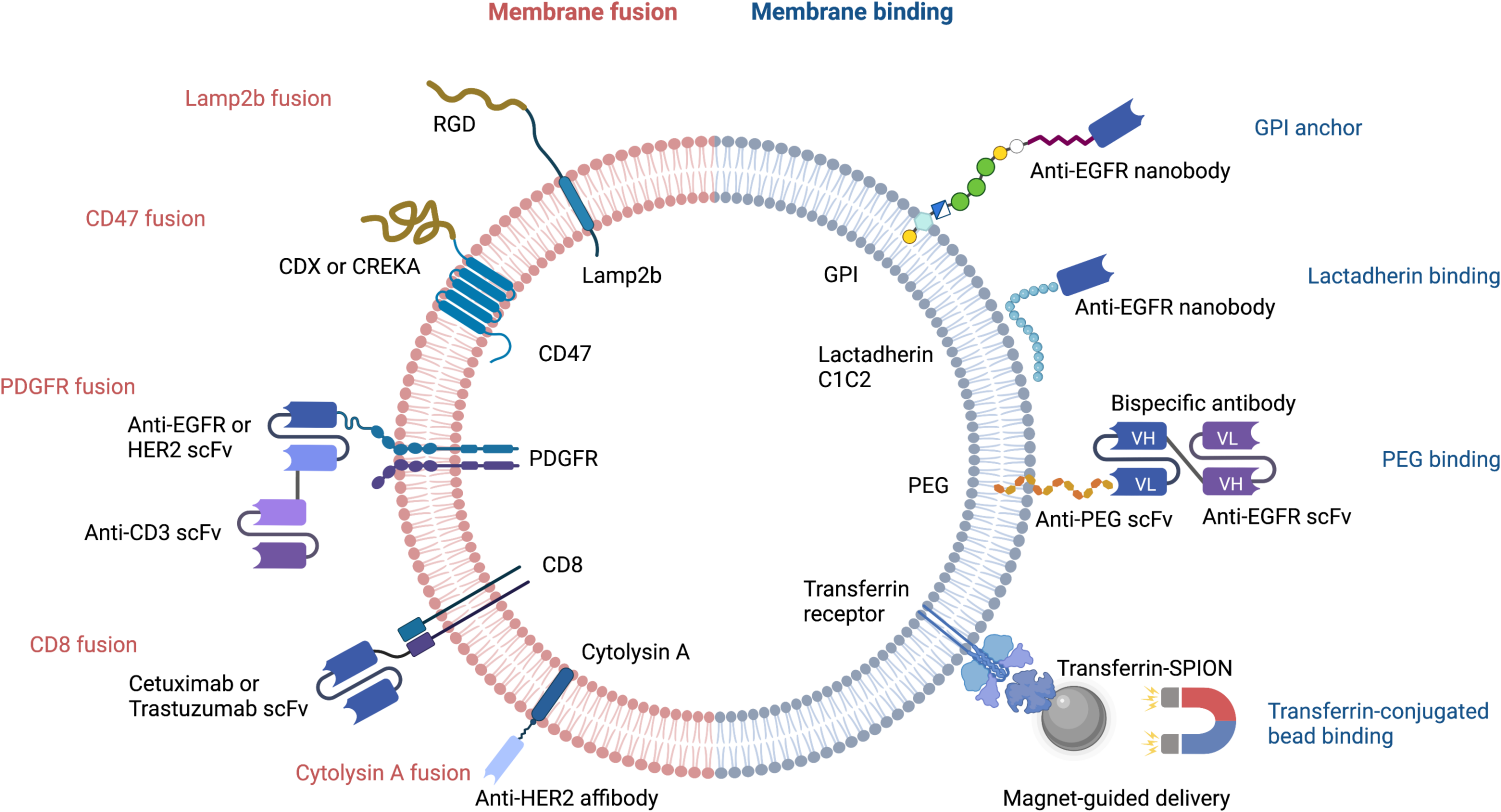
Strategies for targeted delivery of EVs. RGD, Arg-Gly-Asp peptide; CDX, d-peptide ligand of the nicotine acetylcholine receptor; CREKA, pentapeptide Cys-Arg-Glu-Lys-Ala; EGFR, epidermal growth factor receptor; HER2, human epidermal growth factor receptor 2; scFv, single-chain variable fragment; PDGFR, platelet-derived growth factor receptor; GPI, glycosylphosphatidylinositol; PEG, polyethylene glycol; Transferrin-SPION, transferrin-conjugated superparamagnetic iron oxide nanoparticle.

Despite their advantageous small size and ability to cross the blood brain barrier, natural EVs require further optimization before they can be used for drug delivery. Major limitations include the lack of target cell specificity and an inability to store sufficient payload quantities. Great strides have been made with regard to improving the loading of heterogenous cargo (19). EV membranes can be decorated with a wide variety of molecules that increase target cell specificity and/or reduce the likelihood of EV destruction by host macrophages during immune surveillance (20, 21). Genetic and non-genetic manipulation can lead to a release of modified EVs by introducing targeting moieties such as bispecific antibody, single-domain antibody (nanobody), single-chain variable fragment (scFv), affibody, or targeting peptide that can bind to molecules on target cells (**Figure 1**).

### Genetic manipulation of parental cells

Cellular nanoporation (CNP) is the method of choice for delivery of nucleic acids, including large mRNAs, to EVs (19). The mRNAs may encode tumor suppressor genes that comprise a therapeutic payload, or may encode fusion proteins that have a tumor cell-targeting domain and a bioactive domain. Portions of naturally occurring EV membrane proteins are often used in these fusion constructs; the N-terminus of Lamp2b and CD47 are two common examples. Peptides containing the Arg-Gly-Asp (RGD) integrin recognition motif have been fused to Lamp2b to increase the tumor homing capability of EVs (**Figure 1**). This has been used successfully to deliver the chemotherapeutic, doxorubicin, to tumor cells (22). In a similar approach, CDX and CREKA, two other tumor-targeting peptides, have been fused to CD47 to facilitate delivery of mRNA encoding the tumor suppressor, PTEN, to glioblastoma cells (19).

The single-chain transmembrane glycoprotein PDGFR has been exploited for breast cancer immunotherapy by Cheng et al., who designed nanoscale controllers termed synthetic multivalent antibody retargeted exosomes (SMART-Ex) (23). Using the PDGFR transmembrane domain as an anchor, single-chain variable fragments (scFv) that recognize either CD3, EGFR, or HER2 have been introduced to EV membranes (23, 24) (**Figure 1**). The CD8 transmembrane region has also been used to generate chimeric antigen receptor (CAR)-expressing EVs that target either EGFR or HER2; the CAR-EVs promoted tumor regression and did not elicit significant toxicity (25).

Interestingly, EV-type structures from non-mammalian organisms are also being evaluated for their potential as drug carriers. A case in point are bacterial outer membrane vesicles that have been engineered to express a HER2-specific antibody fused to the transmembrane region of the bacteria pore-forming protein, cytolysin A (26).

### Modification of EV membranes

The therapeutic efficacy of EVs can be increased by bioconjugation of nanobodies or bispecific antibodies that bind to specific molecules and receptors on target cells. The ExoMAb approach exemplifies this strategy of engineering EVs to express non-natural antigens (27). Briefly, lactadherin contains a C1C2 domain which binds phosphatidylserine moieties in EV membranes. This provides the basis for decorating the EV membrane with exogenous proteins that are fused to C1C2 domain. This has been used to express tumor antigens at high-levels on EV membranes surface antigens, which could be exploited to generate tumor-specific antibodies. Anti-EGFR nanobodies have also been conjugated to glycosylphosphatidylinositol (GPI) anchor signal peptides or to the lactadherin C1C2 domain. This approach led to a remarkable improvement in selective cargo delivery to EGFR-positive cells (**Figure 1**) (28, 29).

**Figure 2:**
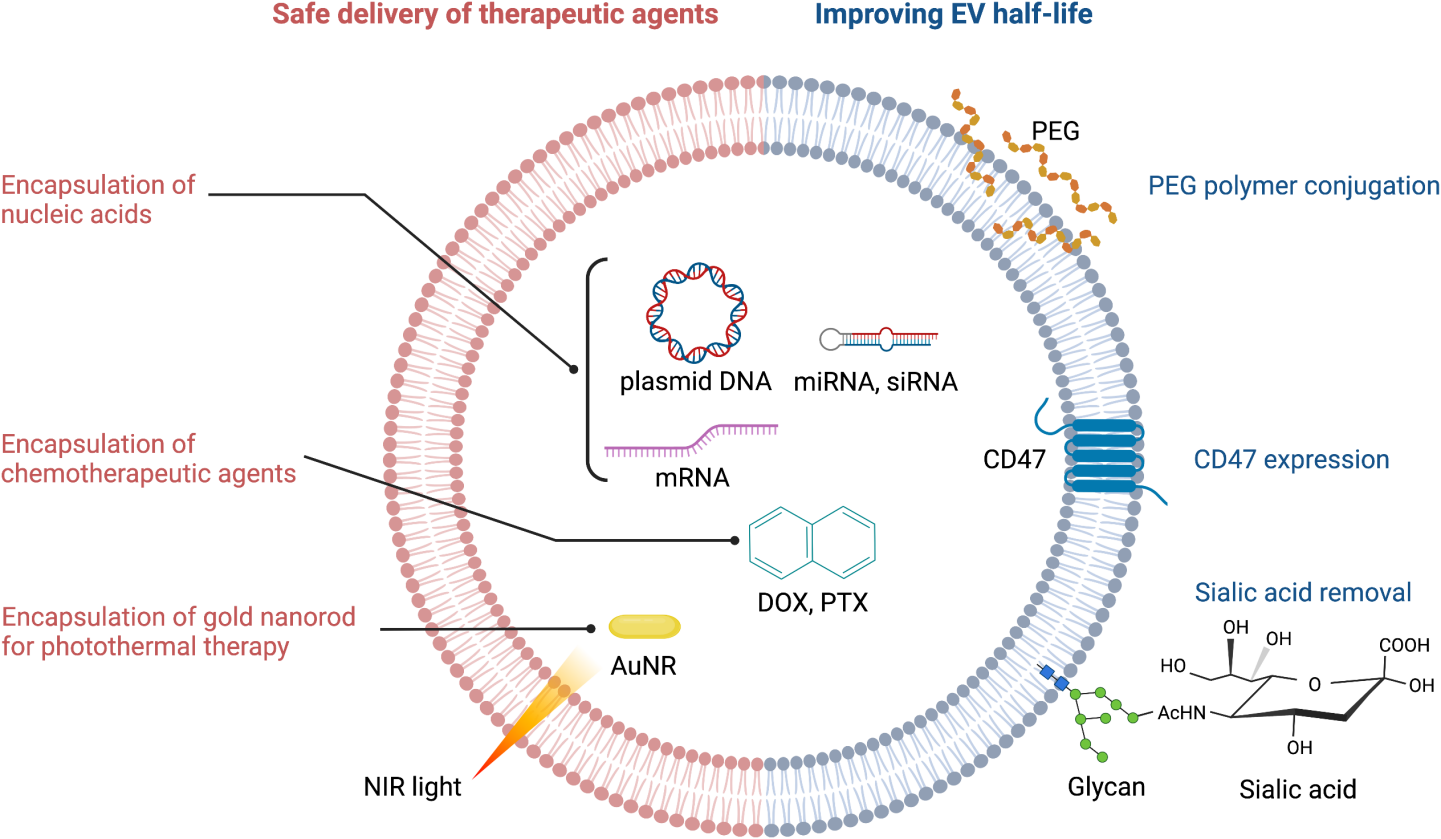
Strategies for improving therapeutic efficacy of EVs. PEG, polyethylene glycol; NIR, near infrared; AuNR, gold nanorod; DOX, doxorubicin; PTX, paclitaxel.

Circulating EVs play a continuous ‘cat and mouse’ game with patrolling macrophages, which can phagocytose EVs upon recognition. This clearly reduces the effective half-life of EVs in the body. CD47 (mentioned above) can again be exploited here: by binding to signal regulatory protein alpha (SIRPα) on the surface of macrophages, CD47 sets up a ‘don’t eat me’ signal (21, 30). Thus, CD47-decorated EVs have longer circulatory half-lives and are more likely to reach their target cancer cells to deliver a therapeutic payload (**Figure 2**). An extremely innovative approach builds on this by combining CD47-loaded red blood cell (RBC) membranes with membranes from cancer cells themselves (31). Since CD47 is overexpressed on the surface of RBC, chimeric EVs derived from RBC-MCF7 hybrid membrane protect EVs from phagocytosis. These hybrid EVs have a longer half-life, but also exploit antigens from the cancer cell membranes to re-target the original cancer cells (31). This has demonstrable therapeutic efficacy, as DOX-loaded hybrid EVs were able to induce tumor regression in a preclinical mouse model.

The EV membrane surface can be chemically or enzymatically modified to either increase EV half-life, or to alter its targeting selectivity. For example, polyethylene glycol (PEG) conjugation to the surface of EVs increases their half-life (32) (**Figure 2**). PEG can also be used as a ‘recognition signal’ for bispecific antibodies. Proof-of-concept is provided with a study using a PEG/EGFR bispecific antibody that increased specific targeting of PEG-loaded EVs to EGFR-positive tumors (33) (**Figure 1**). One can see here that PEG’s effects on EV half-life and antibody targeting is a powerful combination. Altering the surface glycan composition of EVs *via* enzymatic removal of sialic acid moieties also changes their biodistribution (34); this could ultimately be exploited to achieve tumor-specific targeting of EVs (**Figure 2**).

Transferrin receptors are highly enriched on the surface of reticulocyte-derived EVs. This can be exploited to enrich and purify EVs during production, or to facilitate EV-dependent drug delivery. For example, transferrin-conjugated superparamagnetic iron oxide nanoparticles (SPIONs) have been used for targeted delivery of chemotherapeutic drugs (35) (**Figure 1**).

Fusion to liposomes or micelles offers an alternative to chemical modification of EV membranes. For example, fusion of EVs to liposomes using the freeze-thaw method increases the cellular uptake of these hybrid EVs (36). Micelles have been used to deliver PEG-conjugated EGFR single-chain Fab fragment (scFab) to EVs, thereby expanding the spectrum of therapeutics amenable to an EV approach (33).

### Loading of exogenous molecules into EVs

Encapsulation of chemotherapeutic agents and safe delivery to the target tumor are the common challenges in EV-mediated cancer chemotherapy (**Figure 2**). Drugs such as nucleic acids can be loaded *via* passive methods, such as combining drugs with the lipid bilayer after incubation, or conjugating drugs to the EV surface. Alternatively, for drugs with high molecular weight (i.e. DOX, PTX, AuNR), active methods of loading such as mechanical or chemical opening of the EV membrane *via* sonication, electroporation, saponin treatment or freeze-thaw cycles can be employed (37). The active methods create temporary pores through which therapeutic payloads can enter. However, this is not without risk: the internal composition of EVs and their cargos can be compromised, which may reduce efficacy (32). Ultimately, the success of both the passive and active methods is determined by the source of EVs, the specific drug involved, and the experimental design (38).

For the evaluation of EVs in clinical trials, drug loading must be achieved using a robust protocol, and at an industrial scale to meet demands (39). These prerequisites have presented challenges, because EVs lack the intracellular structures of whole cells, and therefore are not amenable to approaches such as conventional transfection.

## Sources of EVs

In this section, we describe the wide variety of systems from which EVs can be sourced. As will become evident, the precise choice of system will depend on the downstream application. We also provide several examples of the application of EVs to cancer therapy.

Despite their substantial contribution to cancer treatment, chemotherapeutic agents are prone to rapid clearance, are poorly biocompatible, and suffer from low intra-tumoral delivery; they also induce systemic side effects and their efficacy is compromised by the onset of drug resistance (40). To overcome these challenges, various types of nanoscale delivery vehicles have been developed. These vehicles are associated with efficient drug delivery and tumor-specific targeting ability, and many have been evaluated in human clinical oncology trials (**Table 1**).

**Table 1:** Clinical trials for EV applications in human cancers.

Phase	Period	Location	N	EV source	EV cargo	Condition	Outcome	Clinical trial number and/or Reference
I	NA - 2002	USA	13	DEX	MAGE tumor antigen	NSCLC	2 stable diseases	(41)
I	2001 - 2004	France	15	DEX	MAGE tumor antigen	Melanoma	2 stable, 1 minor, 1 partial, 1 mixed responses	(42)
I	2006 - 2007	China	40	Malignant ascites	GM-CSF	Colorectal cancer	1 stable disease, 1 minor response	(43)
I	NA - 2008	France	15	DEX	NKG2D ligand	Melanoma	2 tumor regressions	(44)
I	2011 - recruiting	USA	35*	Plant	Curcumin	Colon cancer	NA	NCT01294072
I	2018 - recruiting	USA	28*	MSC	KRAS G12D siRNA	Pancreas cancer	NA	NCT03608631
I	2022 - recruiting	USA	30*	HEK293	STAT6 ASO	Hepatocellular carcinoma, gastric cancer, colorectal cancer	NA	NCT05375604
II	2010 - 2013	France	26	IFNg+ DEX	MAGE tumor antigen	NSCLC	7 stable diseases over 4 months	CSET 2008/1437 IDRCB 2008-A1171-54 (45)
II	2010 - 2015	France	41	DEX	MAGE tumor antigen	NSCLC	Progression free survival over 4 months	NCT01159288
II	2013 - 2014	China	30*	TEX	MTX, Cisplatin, PTX	Malignant pleural effusion, malignant ascites	NA	NCT01854866 (46)
II	2016 - 2019	China	90*	TEX	MTX	Malignant pleural effusion	NA	NCT02657460
NA	2013 - 2015	China	6	TEX	Cisplatin	End stage lung cancer	3 improved pleural effusions	(47)
NA	2015 - 2018	China	20	TEX	MTX	Cholangiocarcinoma	5 reliefs in biliary obstruction	ChiCTR-OIB-15007589 (48)
NA	2015 - 2019	China	62	TEX	MTX	Malignant pleural effusion	Decrease in MPE	ChiCTR-ICR-15006304 (49)

DEX, dendritic cell-derived EVs; MAGE, melanoma-associated antigen; NSCLC, non-small cell lung cancer; GM-CSF, granulocyte-macrophage colony-stimulating factor; ASO, antisense oligonucleotide; IFNg, interferon gamma; MTX, methotrexate; PTX, paclitaxel; NA, not applicable. *Estimated enrollment.

### Tumor-derived EVs (TEX)

Tumor-derived EVs (TEX) preferentially home to and target the tumor cells from which they were derived (50) (**Table 2**). For example, accumulation of DOX in HT1080 xenografts was higher when the drug was delivered by HT1080-derived TEX rather than by those obtained from HeLa cells. Successful inhibition of tumor growth in other preclinical models using TEX carrying a chemotherapeutic payload has been independently confirmed (46). Furthermore, TEX have been used to deliver DOX-loaded porous silicon nanoparticles (PSiNPs) in mouse models of hepatocarcinoma, melanoma, and breast cancer (54). In each of these situations, beneficial attributes of EVs were observed. These included enhanced accumulation of drugs within tumors, more efficient extravasation and tissue penetration and striking anti-tumor activities.

**Table 2:** Applications of TEX in murine tumor models.

EV source	EV cargo	Murine tumor model	Result	Reference
Rat C6 glioma cell line	Tumor peptide	Glioma	Inhibited tumor growth	(51)
Mouse J774A.1	DOX, c-Met binding peptide	Breast cancer	Inhibited tumor growth	(52)
Mouse Hepa1-6, 4T1, Panc02	HMGN1 adjuvant	Hepatocellular carcinoma	Inhibited tumor growth	(53)
Mouse H22	DOX-loaded porous silicon nanoparticles	Hepatocarcinoma, melanoma, breast cancer	Inhibited tumor growth	(54)
Mouse H22, human A2780	MTX, Cisplatin, PTX	Hepatocarcinoma, ovarian cancer	Inhibited tumor growth	(46)
Human HT1080, HeLa	DOX	Fibrosarcoma	Inhibited tumor growth	(50)
Human MCF-7	V_2_C QDs, TAT peptide, PEG, RGD	Breast cancer	NIR laser irradiation almost completely killed tumor cells	(55)
Hybrid RBC and MCF-7 cell membranes	DOX	Breast cancer	Inhibited tumor growth	(31)
Human MDA-MB-231	PTX, CuB	Breast cancer	Inhibited tumor growth and lung metastasis	(56)

DOX, doxorubicin; c-Met, mesenchymal-epithelial transition factor; HMGN1, high mobility group nucleosome-binding protein 1; MTX, methotrexate; PTX, paclitaxel; V_2_C QDs, vanadium carbide quantum dots; TAT, transactivator of transcription of human immunodeficiency virus; PEG, polyethylene glycol; RGD, Arg-Gly-Asp peptide; NIR, near infrared; RBC, red blood cell; CuB, cucurbitacin B.

Glioma is a devastating malignant brain tumor with a high mortality rate due to low penetration and efficacy of the available chemotherapeutics. TEX are already having an impact in this tumor type. For example, TEX-mediated delivery of IMV-001 (an antisense oligonucleotide against the transmembrane receptor, IGF1R) to patients with recurrent malignant glioma was more effective than other delivery methods due as it was associated with an increased number of tumor-infiltrating lymphocytes and enhanced anti-tumor immunity (57, 58). In glioblastoma, co-delivery of TEX with α-galactosylceramide enhanced the efficacy of a dendritic cell vaccine, likely due to its stimulation of immunomodulatory factor release into the tumor microenvironment (51).

In the liver cancer setting, administration of functionalized TEX loaded with an adjuvant, high mobility group nucleosome-binding protein 1 (HMGN1), reduced tumor size and potentiated immunogenicity in a mouse model of hepatocellular carcinoma (53) (**Table 2**). Furthermore, treatment of end-stage extrahepatic cholangiocarcinoma patients with malignant biliary obstruction with MTX-loaded TEX was efficacious and relieved biliary obstruction (48).

Pleural effusions and ascites are abnormal fluid collections within the thoracic and peritoneal cavity, respectively. They are frequent in terminal stage malignancies, and require aspiration and paracentesis to manage disease conditions. TEX may be particularly suited to drug delivery for pleural effusions. For example, A549 tumor cell-derived EVs loaded with methotrexate (MTX) induced neutrophil recruitment in the effusion fluid and improved primary malignant pleural effusion (MPE) in non-small cell lung cancer patients (49). Additionally, two clinical trials have been conducted in patients with MPE to explore the anti-tumor effects of methotrexate- or cisplatin-loaded TEX (NCT01854866, NCT02657460) (**Table 1**). End-stage lung cancer patients with metastatic malignant pleural effusion (MPE) and resistance to cisplatin were treated with cisplatin-loaded TEX (47). This led to a 95% reduction in the number of tumor cells in the malignant fluids, and was associated with increased survival rate without significant side effects.

In addition to tumor cells themselves, TEX are also present in ascites. These ascites-derived TEX can also be exploited for cancer treatment, as exemplified in a clinical trial (43) (**Table 1**). This study demonstrated that treatment of advanced colorectal cancer with TEX from malignant ascites plus GM-CSF induced a specific anti-tumor T cell response.

### Mesenchymal stem cell-derived EVs (STEX)

Mesenchymal stem cell (MSC)-derived EVs (STEX) arise from different tissues in normal and disease conditions and have beneficial effects in wound healing (59), myocardial infarction (60), acute kidney injury (61), hepatic injury (62), neonatal lung injury (63) and optic nerve injury (64). STEX may be particularly suitable for therapeutic applications due to their excellent safety profile, low immunogenicity, and their ability to cross biological barriers (65). They have been used to successfully deliver both chemical and genetic payloads, as we discuss below.

Human umbilical cord STEX enhance drug-induced apoptosis of leukemic cells *in vitro* (66) and increase their sensitivity to the anticancer drug imatinib through caspase activation (67). STEX can also deliver genetic cargos. For example, STEX-mediated delivery of miR-379 significantly suppressed breast cancer growth *in vivo* by decreasing cyclooxygenase (COX)-2 expression (68) (**Table 3**). Furthermore, an ongoing phase I clinical trial in KRAS G12D-mutant pancreatic cancer is evaluating the optimal dose and adverse effects of STEX loaded with KRAS G12D siRNA (NCT03608631) (**Table 1**).

**Table 3:** Applications of MSC- and HEK293-derived EVs in murine tumor models.

EV source	EV cargo	Murine tumor model	Result	Reference
Mouse MSCs	TNFα, SPION	Melanoma	Inhibited tumor growth	(69)
Rat AD-MSCs	b-catenin	Hepatocellular carcinoma	Inhibited tumor growth	(70)
Mouse AD-MSCs	miR-16-5p	Breast cancer	Inhibited tumor growth	(71)
Human AD-MSCs	miR-101	Osteosarcoma	Inhibited lung metastasis	(72)
Human BM-MSCs	GRP78 siRNA	Hepatocellular carcinoma	Inhibited tumor growth and metastasis	(73)
Human BM-MSCs	miR-379	Breast cancer	Inhibited tumor growth	(68)
Human HEK293	ASO-STAT6	Colorectal cancer, hepatocellular carcinoma	Inhibited tumor growth	(74)
Human HEK293T	BCR-ABL siRNA, Imatinib, LAMP2B-IL3	Chronic myeloid leukemia	Inhibited CML	(75)
Human HEK293T	PSMA, EGFR, or Folate aptamer; Survivin siRNA	Prostate, breast, colorectal cancer	Inhibited tumor growth	(76)
Human HEK293T	DOX, lipHA	Breast cancer	Inhibited tumor growth	(77)
Human HEK293T	miR-21-sponge	Glioma	Inhibited tumor growth	(78)
Human HEK293T	miR-21 inhibitor, 5-FU, LAMP2-HER2 affibody	Colorectal cancer	Inhibited tumor growth	(79)

SPION, superparamagnetic iron oxide nanoparticle; GRP78, glucose regulatory protein 78; ASO-STAT6, antisense oligonucleotide targeting STAT6; BCR-ABL, fusion gene of breakpoint cluster region BCR and tyrosine-protein kinase ABL1; LAMP2B-IL3, fusion protein of LAMP2B and interleukin 3; CML, chronic myeloid leukemia; PSMA, prostate-specific membrane antigen; EGFR, epidermal growth factor receptor; lipHA, lipidomimetic chains-grafted hyaluronic acid; LAMP2-HER2 affibody, fusion protein of LAMP2 and anti-HER2 (human epidermal growth factor receptor 2) affibody.

Adipose tissue-derived mesenchymal stromal cells (AD-MSCs) are one of the most studied STEX sources because they are easier to obtain and harvest through subcutaneous lipoaspiration; this is much less painful and less ethically sensitive than collecting bone marrow or embryonic stem cells (80, 81). In the first animal study of its kind, Ko et al. found that AD-MSC-derived EVs significantly increased the number of circulating NKT cells and inhibited HCC tumor growth (70). Following demonstrations that AD-MSCs have anti-tumor effects in bladder cancer (82), prostate cancer (83) and breast cancer (71) *via* induction of apoptosis, there has been a surge of interest in the use of AD-MSCs for cancer therapy. This is highlighted by the finding that delivery of miR-101 by AD-MSC-derived EVs downregulated BCL6 and inhibited the metastasis of osteosarcoma to the lungs in a preclinical mouse model (72) (**Table 3**).

Bone marrow-derived STEX carrying GRP78 siRNA enhanced sorafenib sensitivity in an HCC xenograft mouse model (73) (**Table 3**). Moreover, miR-125b-loaded EVs derived from AD-MSCs specifically reduced HCC cell proliferation *in vitro* by activation of the p53 tumor suppressor (84). STEX are currently under investigation in clinical trials beyond cancer, including those focused on neurological and cardiovascular diseases, and SARS-CoV-2 (85).

### HEK293-derived EVs (HEX)

HEK293T cells have been widely used as EV producer cells due to their inherent rapid proliferation, high EV yield, and ease of genetic manipulation (86–90). HEK293-derived EVs (HEX) have been used to deliver gene therapies including miRNA for breast cancer (91) and both chemotherapeutics and therapeutic protein constructs in a schwannoma model (92). Notably, HEX are more readily taken up by human neural stem cells when compared to mature neurons, suggesting that they might be used to modulate undifferentiated neurons in future therapeutic applications (93).

Administration of HEX loaded with a miR-21 sponge construct (which prevents miR-21 binding to its natural target) significantly reduced tumor burden in a C6 glioma rat model (78) (**Table 3**). Since miR-21 is overexpressed in glioma, we infer that miR-21 sponge-loaded EVs will reduce the proliferation and malignant metastatic behavior of tumor cells in patients. Additionally, HEX loaded with an antisense oligonucleotide (ASO) targeting STAT6 selectively silenced expression of this transcription factor in colorectal cancer and HCC mouse models; combined with anti-PD1 immunotherapy, this led to greater than 90% inhibition of tumor growth (74) (**Table 3**). With the same strategy, an ongoing phase I clinical trial is evaluating the pharmacokinetics and pharmacodynamics of exoASO-STAT6 (CDK-004) in advanced HCC, colorectal cancer, and liver metastases from gastric cancer (NCT05375604) (**Table 1**).

### Dendritic cell-derived EVs (DEX)

Cancer vaccines are used to boost the endogenous human immune response to cancer through enhanced recognition of tumor cell-related antigens. Adding to the therapeutic arsenal in this area are dendritic cell-derived EVs (DEX), which express MHC-I- and MHC-II-bound antigen peptides as well as other adhesion molecules (94). Pioneering work demonstrated that EVs derived from tumor peptide-pulsed bone marrow dendritic cells (BMDC) could prime cytotoxic T cells and thereby facilitate tumor growth inhibition in syngeneic tumor mouse models (95) (**Table 4**).

**Table 4:** Applications of DC-, NK-, and macrophage-derived EVs in murine tumor models.

EV source	EV cargo	Murine tumor model	Result	Reference
Mouse BMDCs	Tumor peptide	Mastocytoma, mammary adenocarcinoma	Inhibited tumor growth	(95)
Mouse imDCs	DOX, Lamp2b-RGD	Breast cancer	Inhibited tumor growth	(22)
Human NK92-MI	FasL, perforin, TNFa	Melanoma	Inhibited tumor growth	(96)
Human NK cells from healthy donor	NKG2D, NKp44, NKp30	Neuroblastoma	Inhibited tumor growth	(97)
Human NK cells from healthy donor	miR-186	Neuroblastoma	Inhibited tumor growth	(98)
Mouse RAW264.7	PTX	Lung cancer	Inhibited tumor growth and lung metastasis	(99)
Mouse RAW264.7	LFA-1	Colorectal cancer	Inhibited tumor growth	(100)
Mouse RAW264.7	DOX	Breast cancer	Inhibited tumor growth	(101)

BMDC, bone marrow-derived dendritic cell; imDC, mouse immature dendritic cell; RAW264.7, mouse macrophage cell line; Lamp2b-RGD, fusion protein of Lamp2b and Arg-Gly-Asp peptide; LFA-1, lymphocyte function-associated antigen 1.

DEX can activate CD4+ T cells by inducing Th1 and Th2 immune responses, irrespective of the maturity of the DCs (102). However, large and small DEX constructs differ in their capacity to induce a T cell response, favoring cytokine secretion by Th2 and Th1 cell subtypes, respectively. A Phase II clinical trial evaluating DEX loaded with tumor antigen in patients with unresectable non-small cell lung cancer has recently been completed. The trial revealed activation of both innate and adaptive immunity, and over 4 months of progression-free survival in 32% of patients (NCT01159288) (45) (**Table 1**). Another Phase I trial in this cancer type showed that DEX therapy was well-tolerated and elicited only minor adverse effects (41).

### NK cell-derived EVs (NEX)

NK cell-derived EVs (NEX) can carry cargo such as cytotoxic proteins, miRNAs, and cytokines that employ multiple mechanisms to kill tumor cells (103). They also exhibit immunomodulatory activity by stimulating other immune cells. Prior studies have demonstrated that NEX contain not only FasL and perforin but also TNFα; these molecules can all trigger melanoma cell death (96, 104) (**Table 4**). NEX exposed to neuroblastoma cells can ‘teach’ naive NK cells to recognize and kill these cancer cells, thereby overcoming immune resistance (97). Moreover, tumor cell growth is blocked by NEX containing the tumor suppressor miR-186, and these NEX also derepress TGFβ1-dependent inhibition of NK cells (98). These findings suggest that, in addition to their role as drug carriers, NEX can act as immunotherapeutic agents.

### Macrophage-derived EVs (MEX)

Macrophages can be categorized into anti-tumor M1 and protumor M2 subtypes, and reprogramming between these phenotypes has been exploited for anticancer therapy (74). Macrophage-derived EVs (MEX) derived from the M1 macrophage cell line, RAW264.7, accumulate at the tumor site and promote M2-to-M1 macrophage reprogramming (100). MEX loaded with DOX also induced apoptosis and suppressed the growth of xenografts in a murine model of breast cancer (101) (**Table 4**).

### Plant-derived EVs (PEX)

Fruits and vegetables have been used historically as medicines to treat numerous diseases. Following the discovery of plant-derived EVs (PEX), which can be isolated by extracting apoptotic vesicles from leaf, sunflower seeds, and roots (105), it was a natural progression to investigate whether they could be harnessed for therapeutic benefit (106). The choice of a specific PEX is determined by the type of health-promoting molecule that is enriched within each plant; this selection must match the disease to be treated. Specific examples follow below.

The consumption of grapes may reduce the impact of risk factors associated with multiple diseases, including cancer (107). This has been attributed to the presence of anti-inflammatory flavonoids (107), phenolic acids and polyphenols that exert anti-cancer activity by scavenging reactive oxygen species (108). It is therefore not surprising that PEX from grapes and grapefruit exhibit anti-inflammatory properties (109, 110) and can reduce colitis in murine models (109, 111).

Ginger contains gingerol, which has powerful antioxidant and anti-inflammatory properties (112); together with other compounds in ginger root, this may explain the ability of extracts from this plant to inhibit oxidative stress, arthritis, inflammation, and various types of infection (113). Ginger may also decrease the risk of cancer and diabetes (112, 114). As highlighted for grapes, ginger PEX are also able to attenuate inflammation (115) and reduce the pathologies associated with colitis (116).

Although their anti-inflammatory effects are well established, the evaluation of PEX as cancer therapeutics is still in the early stages. However, initial results are encouraging. For example, PEX from citrus lemons suppressed chronic myeloid leukemia xenograft growth by inducing TRAIL-mediated cell death (117) (**Table 5**). Additionally, an ongoing phase I clinical trial (NCT01294072) is evaluating whether PEX can be used to deliver curcumin (a constituent of the spice, turmeric) to perturb tumor cell metabolism and modulate the immune response in colon cancer patients (**Table 1**). There is a strong underlying preclinical evidence for this, because curcumin was reported to suppress colon cancer in a murine xenograft model by inhibiting Wnt/β-catenin signaling (122). A separate *in vitro* study showed that curcumin significantly arrested the growth of human colon cancer cells (123). These promising early data provide the rationale for further study of the therapeutic effects of PEX in diverse diseases.

**Table 5:** Applications of plant-derived and other EVs in murine tumor models.

EV source	EV cargo	Murine tumor model	Result	Reference
Grapefruit	Stat3 inhibitor JSI-124	Brain cancer	Inhibited tumor growth	(118)
Citrus limon	TRAIL-stimulating factor	Chronic myeloid leukemia	Inhibited CML	(117)
Mouse whole blood	Photosensitizer PpIX fused with NLS peptide	Breast cancer	630 nm He-Ne laser irradiation inhibited tumor growth	(119)
Human neutrophils from healthy donor	DOX, SPION	Colorectal cancer	Inhibited tumor growth	(120)
Human BJ foreskin fibroblast	KRAS G12D siRNA	Pancreatic cancer	Inhibited metastasis	(21)
Human H9 ES cell line	PTX, RGD	Brain cancer	Inhibited tumor growth	(121)

PpIX, Protoporphyrin IX; NLS, nuclear localization signal.

## Application of EVs in cancer therapy

Following the discussion of the sources of EVs above, we now present several examples of how EVs are being used to complement and enhance current anti-cancer approaches. It should become apparent from this discussion that the targeting potential of EVs, as well as some of their innate abilities to penetrate cell membranes, offers great promise in the realm of cancer therapy.

### Tumor-specific targeting

A major obstacle to effective cancer chemotherapy is a lack of tumor-specific targeting, which is associated with toxicity in normal tissues. Superparamagnetic iron oxide nanoparticles (SPIONs) have great potential utility as magnetic nanoplatforms for targeted drug delivery (124), as they can be directed to the required tissue area through the use of external magnets. SPIONs conjugated with transferrin can readily be attached to EVs, which express high surface levels of the transferrin receptor (125). The SPION-decorated EVs can be loaded with a chemotherapeutic agent and then directed to the tumor site using magnets. This has been elegantly demonstrated by Zhang et al., who observed DOX-dependent tumor regression and increased survival in a preclinical model using this approach (120). The SPION approach has also been used to enhance the cytotoxic effect of the pro-apoptotic cytokine, TNFα, in melanoma cells (69).

An alternative to SPIONs with regard to tumor-specific targeting is the use of aptamers. Aptamers are short single-stranded DNA or RNA that can bind to a specific target molecule (126). Aptamers can be used for therapeutic purposes in the same way as monoclonal antibodies. For example, EVs loaded with siRNA against the anti-apoptotic molecule, survivin, have been specifically delivered to prostate cancer cells *in vivo* using prostate-specific membrane antigen (PSMA) aptamers (76) (**Table 3**). In a similar manner, EGFR aptamer-conjugated EVs loaded with survivin siRNA inhibited tumor growth in an orthotopic breast cancer mouse model (76). We believe that the aptamer approach could readily be exploited to treat other cancer types. For example, the interleukin-3 receptor (IL3R) is overexpressed on chronic myeloid leukemia (CML) cells, but is absent or expressed at low levels on normal hematopoietic stem cells. This suggests that IL3R could serve as a receptor target for EV-based cancer drug delivery systems (127–129).

### Overcoming drug resistance

Following several rounds of chemotherapy, the onset of drug resistance leads to reduced survival. Several lines of evidence suggest that EVs may offer breakthroughs in this challenging area. For example, HEX modified with lipidomimetic chain-grafted hyaluronic acid can efficiently deliver DOX and reverse multi-drug resistance in breast cancer cells (77) (**Table 3**). In a 5-FU-resistant HER2-positive colorectal cancer mouse model, co-delivery of an miR-21 inhibitor using EVs decorated with a LAMP2-HER2 affibody fusion significantly enhanced cytotoxicity and restored 5-FU sensitivity (79). Multidrug-resistant Madin-Darby canine kidney cells can be re-sensitized to paclitaxel when the drug is delivered *via* EVs (99). Finally, HEX loaded with imatinib and siRNA to the driver oncogene, BCR-ABL, have been used to re-sensitize CML xenografts to imatinib in a preclinical model (75).

### Hard-to-treat tumors

Primary brain tumors and tumors that metastasize to the brain are difficult to treat due to poor drug penetrance across the blood brain barrier (BBB). EVs may offer a natural solution to this problem because they are nanosized membrane vesicles that can easily pass through the BBB. The combination of BBB penetration and tumor cell-selective targeting using EVs has been compellingly demonstrated in glioma. Specifically, EVs decorated with RGERPPR, a peptide ligand for neuropilin-1 which is highly expressed on glioma cells but not other neuronal cells (130, 131), were able to target glioma cells in an orthotopic mouse model (132). Another example of EV success in brain tumors is the selective targeting of glioblastoma cells, which overexpress integrin αVβ3 receptors (133). EVs loaded with paclitaxel (PTX) and conjugated with RGD peptides which have high affinity for αVβ3 receptors were able to target orthotopic glioblastomas and prolong survival in a mouse model (121).

Triple negative breast cancer (TNBC) has the worst prognosis of all breast cancers, primarily because it lacks estrogen and progesterone receptors, and has extremely low levels of HER2 (134, 135). However, TNBC cells express high levels of the c-Met tyrosine kinase oncogene, which is associated with poor prognosis and drug resistance (136, 137), and could be targeted by EVs. Proof-of-concept has been demonstrated *via* the delivery of DOX to TNBC xenografts using MEX coated with the c-Met binding peptide, CBP (52, 138).

### Enhancing other therapeutic modalities

EVs can be used in combination with cutting edge drug delivery and activation technologies, such as photothermal and photodynamic therapies. Gold nanorods (AuNRs) have unique properties that make them attractive for applications in bioimaging, drug delivery, and photothermal therapy in cancer (139). Irradiating AuNRs with near-infrared light (NIR) produces a moderate temperature rise in the target region that leads to selective damage in tumor tissues (**Figure 2**). Since NIR lasers can be manipulated precisely, this activation mechanism provides an ideal and versatile platform to simultaneously deliver heat and anticancer drugs with control over the exposure area, time, and dosage. An alternative photothermal agent is the vanadium carbide quantum dot (V_2_C QD). This compound has higher photothermal conversion efficiency than AuNRs, and V_2_C QD-loaded EVs have superior biocompatibility and long circulation times combined with advanced antitumor activity (55) (**Table 2**).

Photodynamic therapy (PDT) requires light and a photosensitizing chemical substance that produces an oxygen radical to elicit cell death (140). EVs conjugated with the photosensitizer protoporphyrin IX (PpIX) and nuclear localization signal (NLS) peptide have good biocompatibility and the ability to target the nucleus. This subcellular targeting strategy and the cytotoxic reactive oxygen species generated by the photosensitizer enhances the efficacy of PDT, opening a new window for cancer therapy (119).

## Conclusion and future perspective

There is great demand for safe, efficient, and versatile drug delivery systems for cancer therapy. Engineered EVs hold great promise in this regard, given their ability to specifically target and efficiently transfer therapeutic agents into cancer cells. However, more evaluation is required to produce and incorporate engineered EVs into clinically relevant systems, weighing the potential risks and benefits of this new approach. While the use of allogenic EVs appears feasible, the selection of parental cells, assessment of immunologic and oncogenic effects, and risk of viral contamination need to be carefully considered.

Successful clinical translation of EVs depends on the availability of reliable methods for large-scale production, isolation, and characterization to minimize lot-to-lot variations in drug-loaded EVs (141). A potential solution to many of these challenges is the use of EV mimics or artificial EV generated by chemical or genetic modification. Fusion of drug-loaded liposomes with EVs can enhance drug loading and targeting abilities (142), while customized production of EVs by exogenously implanted cells offers a new route for the production of engineered EVs *in vivo* (143). Although further studies are required to develop safer, more efficient, and cost-effective methods for generating engineered EVs for practical applications in oncology, the future is decidedly bright for this next-generation cancer therapy.

## Author contributions

The author confirms being the sole contributor of this work and has approved it for publication.
